# Synthesis of Highly Luminescent Silica-Coated Upconversion Nanoparticles from Lanthanide Oxides or Nitrates Using Co-Precipitation and Sol–Gel Methods

**DOI:** 10.3390/gels10010013

**Published:** 2023-12-22

**Authors:** Ana Iglesias-Mejuto, Alyne Lamy-Mendes, João Pina, Benilde F. O. Costa, Carlos A. García-González, Luisa Durães

**Affiliations:** 1 AerogelsLab, I + D Farma Group (GI-1645), Department of Pharmacology, Pharmacy and Pharmaceutical Technology, Faculty of Pharmacy, iMATUS and Health Research Institute of Santiago de Compostela (IDIS), Universidade de Santiago de Compostela, E-15782 Santiago de Compostela, Spain; 2 University of Coimbra, CIEPQPF—Centro de Investigação em Engenharia dos Processos Químicos e Produtos da Floresta, Department of Chemical Engineering, 3030-790 Coimbra, Portugalluisa@eq.uc.pt (L.D.); 3 Coimbra Chemistry Centre—Institute of Molecular Sciences, Department of Chemistry, University of Coimbra, 3004-535 Coimbra, Portugal; jpina@qui.uc.pt; 4 University of Coimbra, CFisUC, Physics Department, 3004-516 Coimbra, Portugal; benilde@ci.uc.pt

**Keywords:** upconversion luminescence, co-precipitation method, sol–gel Stöber method, lanthanide nitrates, lanthanide oxides

## Abstract

Upconversion nanoparticles (UCNPs) are under consideration for their use as bioimaging probes with enhanced optical performance for real time follow-up under non-invasive conditions. Photostable and core-shell NaYF_4_:Yb^3+^, Er^3+^-SiO_2_ UCNPs obtained by a novel and simple co-precipitation method from lanthanide nitrates or oxides were herein synthesized for the first time. The sol–gel Stöber method followed by oven or supercritical gel drying was used to confer biocompatible surface properties to UCNPs by the formation of an ultrathin silica coating. Upconversion (UC) spectra were studied to evaluate the fluorescence of UCNPs upon red/near infrared (NIR) irradiation. ζ-potential measurements, TEM analyses, XRD patterns and long-term physicochemical stability were also assessed and confirmed that the UCNPs co-precipitation synthesis is a shape- and phase-controlling approach. The bio- and hemocompatibility of the UCNPs formulation with the highest fluorescence intensity was evaluated with murine fibroblasts and human blood, respectively, and provided excellent results that endorse the efficacy of the silica gel coating. The herein synthesized UCNPs can be regarded as efficient fluorescent probes for bioimaging purposes with the high luminescence, physicochemical stability and biocompatibility required for biomedical applications.

## 1. Introduction

Bioimaging enables the tracking of biological functions in real time by using technologies such as X-ray, magnetic resonance or fluorescence for detecting biological probes employed for the monitoring and examination of anatomy and physiology [1]. Namely, optical sensing is one of the most commonly used technologies for biodetection due to its sensitivity and simplicity, but efficient labeling molecules are needed for these purposes [2]. Among them, fluorescent dyes and semiconductor quantum dots (QDs) are being used as bioimaging tools, but the photobleaching of organic dyes, toxicity, poor photostability and broad emission of QDs limit their performance [3]. Moreover, fluorescent probes are generally excited by ultraviolet light, which induces DNA damage and cell death [4]. Thus, the development of materials with improved and highly efficient photochemical performance using biologically tolerable exciting radiation sources is required for in vivo bioimaging applications.

Upconversion nanoparticles (UCNPs) are considered as the new generation of fluorophores due to their unique optical properties such as sharp emission bands, large anti-Stokes shifts and low photobleaching and blinking [5]. These nanocomplexes also present good signal-to-noise ratio and sensitivity due to the low scattering, autofluorescence and absorption from living tissues (background) in the red/near infrared (NIR) region (therapeutic window or NIR window) while presenting high tissue penetration depth (up to 2 mm) [2,5,6,7]. Upconversion (UC) luminescence is an “anti-Stokes emission”, where the emitted fluorescence (visible light from 400 to 700 nm) has shorter wavelengths and higher energy than the incident light, NIR (from 808 to 980 nm), which is a radiation usually regarded as not biologically harmful [8,9] at short exposure levels and with good transmittance through tissues [10,11,12]. The absorption of low-energy photons leads to the emission of high-energy photons by a non-linear UC process that enables the photomediated biosensing and bioimaging in a non-invasive process [13]. Based on this principle, UCNPs present long photoluminescence lifetimes, as well as low toxicity and photodamage for living organisms [3,14]. Indeed, UCNPs allowed the transformation of fluorescence imaging from microscopic to macroscopic levels with potential applications in bioimaging, biodetection, and cell and tissue labeling [4,10]. Moreover, UCNPs are one of the most promising luminescent probes for monitoring whole-body small animals, but in vivo UC luminescence still has some limitations [15]. Among them, sensitivity and resolution are affected by the absorption and scattering effects of organs with a rapid decrease in the signals detected from in vivo experiments involving UCNPs [16]. New luminescence imaging tools based on UCNPs must be developed to address all these concerns.

UCNPs are composed of a host matrix doped with a sensitizer (light absorber) and an activator (light-emitter) as guest molecules [17]. NaYF_4_ nanocrystals are the most efficient UC host materials to date, being detected by confocal microscopy with a size of 27 nm [18] due to the low phonon energy and good absorption [19,20]. The NaYF_4_ lattice forms two crystalline phases, the cubic (α phase) and the hexagonal (β phase) ones [8]. Thermodynamically stable hexagonal phase yields bright luminescence, but the polymorphic change from the cubic to the hexagonal phase requires high energy, e.g., hydrothermal or solvothermal treatment [21]. A decrease in particle size may also cause a decrease in emitter numbers and a high surface quenching [18]. Therefore, the process parameter control in the UCNPs synthetic methods should allow the tuning of the particle size and shape, nanocrystal phase, and doping concentration to ensure effective properties for bioimaging [19]. Regarding the ideal sensitizers, they should have the same NIR excited energy state as activators. Specifically, rare-earth doped UCNPs are photostable and have low photoblinking and photobleaching [12,22]. Namely, ytterbium (Yb^3+^) is used as a sensitizer due to its high absorption and broad excitation spectrum. Its single excited state matches with activators like erbium (Er^3+^), so Yb^3+^ is commonly employed as a sensitizer of Er^3+^. These types of UCNPs contain thousands of emission center ions and can be of interest for bioimaging as they can be excited with NIR, thus enabling the deep penetration in tissues with non-invasive treatments and a low interference with the weak autofluorescence from the biological background [12,23]. It is important to highlight that luminescence intensity and efficiency are strongly dependent on the crystal phase and morphology of the UCNPs, so an environmentally friendly synthetic approach able to yield non-toxic nanosystems with a controlled shape and phase is required [24,25].

Lanthanide-doped UCNPs are usually obtained by a thermal decomposition, hydrothermal or solvothermal method, sol-gel processing or a co-precipitation approach [17]. Thermal decomposition consists of pyrolyzing the organometallic precursors with an organic solvent in an oxygen-free environment [26]. Hydrothermal synthesis is carried out with lanthanide nitrates or chlorides as precursors and under a high pressure and temperature environment, often above the critical point of the solvent [19,21,26]. The sol–gel method starts with a solution of precursors that forms a gel by hydrolysis and polycondensation [4]. After calcination at high temperatures, highly crystalline UCNPs are synthesized, but this strategy is not used for biomedical applications because of the lack of control of particle size [19]. In contrast, the co-precipitation method consists of the simultaneous and rapid precipitation of multiple species under supersaturation conditions in a short time [27]. This UCNPs processing method is regarded as advantageous since, in contrast to the previously mentioned techniques, it does not require complex procedures or harsh conditions (such as high temperatures or the use of solvents), while the obtained UCNPs are chemically homogeneous, and easily obtained with high yields after short reaction times [16,21].

UCNPs with biocompatible surface properties and uniform morphologies are prone to be employed as bio-labels, so ligands like polyethylene glycol, oleic acid or silica are used to functionalize UCNPs, to increase water solubilization and stability and to modulate cellular interactions [4,14,17,28]. Silica modification is a well-established and commonly employed technique, easy to obtain, effective and able to improve the biocompatibility and stability of the resulting structures [7]. By the sol–gel Stöber method [29], proposed in 1968 by Werner Stöber and coworkers, TEOS (alkoxide precursor) is hydrolyzed to produce silicic acid, which undergoes a condensation reaction in mild basic conditions to form an amorphous silica coating around the previously synthesized lanthanide core, in the presence of ammonia as a catalyst [30,31]. This eco-friendly alternative sol–gel method avoids the use of potentially cytotoxic surfactants and organic solvents that would require several washing steps prior to be employed as biological labels. Moreover, the ultrathin silica shell synthesized is non-toxic, chemically inert, and optically transparent, which are important advantages to render photoluminescent UCNPs for bioimaging applications [32].

In this work, core-shell NaYF_4_:Yb^3+^, Er^3+^-SiO_2_ UCNPs obtained from lanthanide nitrates and, alternatively, from lanthanide oxides, were synthesized. A novel and simple co-precipitation method was applied, followed by a silica coating step based on TEOS hydrolysis by the sol–gel Stöber process. The obtained UCNPs formulations were characterized regarding their crystalline structure and photophysical properties by XRD, SEM-EDS and UC fluorescence spectroscopy. TEM and confocal microscopy were performed to unveil the morphological and luminescent properties of UCNPs just after synthesis and, comparatively, after a 2-year mid-term storage. Cell viability tests with murine fibroblasts and hemolytic activity tests with human blood were also performed for biocompatibility evaluation. Lanthanide-derived core-shell NaYF_4_:Yb^3+^, Er^3+^-SiO_2_ UCNPs yielded biocompatible nanocomplexes with high crystallinity, a controllable transition from cubic α-NaYF_4_ to hexagonal β-NaYF_4_ and long-term UC fluorescence emission and physicochemical stability. To the best of our knowledge, it is the first time that silica gel-coated UCNPs were synthesized using a procedure involving lanthanide nitrates or oxides as precursors, in a room-temperature (RT) facile, versatile, and non-hazardous co-precipitation method. Also, precursors were dissolved in aqueous solutions, thus avoiding the use of solvents or surfactants that could render toxic by-products. Overall, the novel technological combination of co-precipitation and sol–gel methods herein studied is regarded as environmentally benign and very convenient for obtaining highly photoluminescent UCNPs in a shape- and phase-controlling approach.

## 2. Results and Discussion

### 2.1. Physicochemical Properties of UCNPs

Different formulations of nanoparticles (NPs)were obtained (Table 1) and denoted differently depending on the lanthanide precursors (lanthanide nitrates—N or lanthanides oxides—O), drying procedure (supercritical drying—SCD, or oven drying—OD) and when the drying was performed (before or after the silica coating—BC or AC, respectively). A formulation without coating (O, OD) was also obtained for the sake of particle size and morphology comparison.

All the tested synthetic procedures led to the production of UCNPs formulations in the form of a whitish and fine powder. Similar yields were observed for all the studied methods, with a slightly lower amount of UCNPs obtained after procedures involving supercritical drying (SCD) due to some mass losses during the drying step. Synthesis was simpler in the case of the use of nitrates of the lanthanides than in the case of oxides of the lanthanides due to the pH adjustment needed in the latter case. Near-zero and negative zeta potential (ζ-potential) values were obtained for all the formulations studied (Table 2). These values are good since highly negative charged NPs could be passively delivered to certain tissues [33] while highly positive charged NPs induce cell electrostatic interactions that increase endocytic uptake and are thus more toxic [4,34].

**Figure 1:**
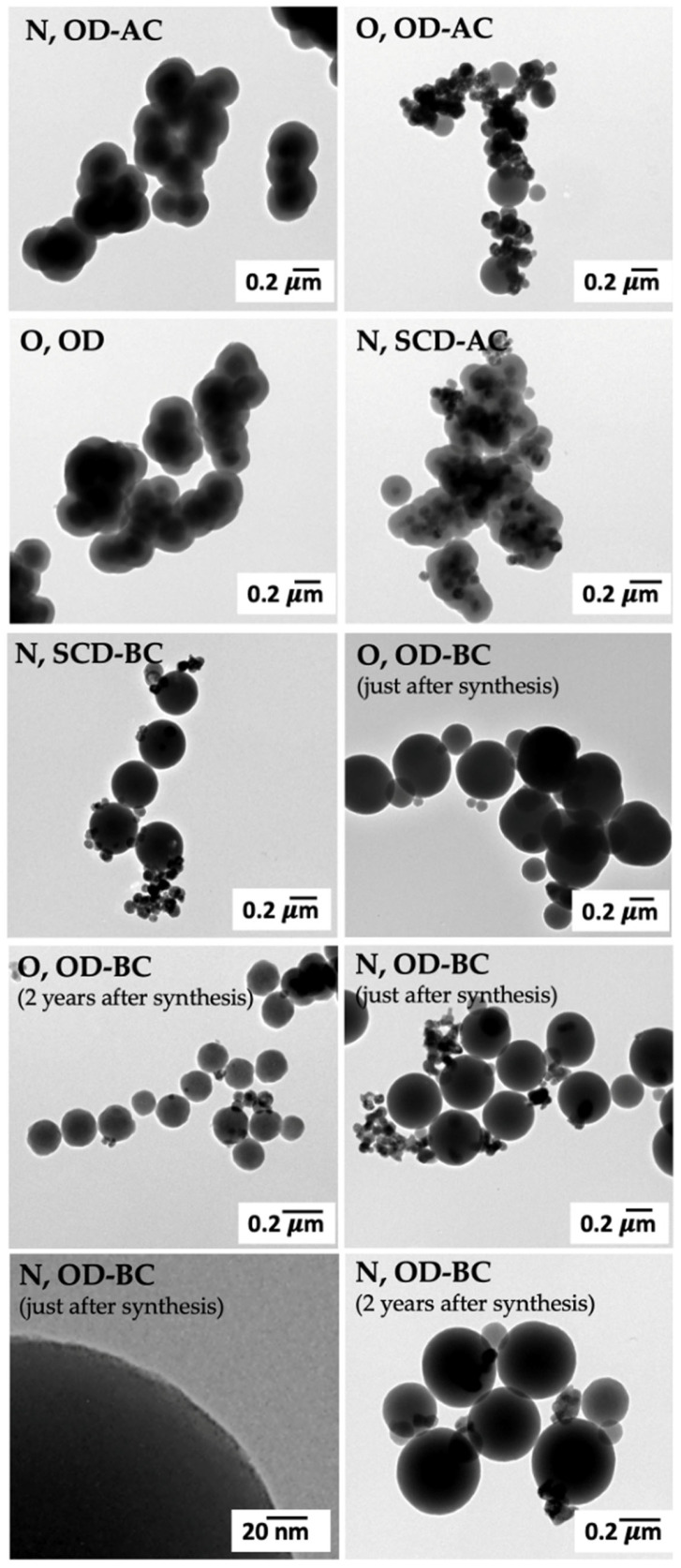
TEM images of different UCNPs formulations. For notation of the formulations, please refer to Table 1.

The formation of UCNPs with uniformity in shape was observed by TEM imaging (Figure 1) in all cases, regardless of the gel drying method used (SCD or oven drying) or the silica coating (presence or absence). This is a very good result since all the formulations are highly spherical (Figure 1), thus demonstrating the shape-controlling ability of the co-precipitation approach herein employed. The two formulations dried by SCD (Figure 1 N, SCD-AC; N, SCD-BC) had an aspect and size close to that of the oven-dried UCNPs (Figure 1 N, OD-AC; O, OD-AC; O, OD; O, OD-BC; N, OD-BC), suggesting the absence of an impact of the drying procedure employed during synthesis in the morphology of UCNPs. It should also be noted that the formulation without a silica coating (Figure 1 O, OD) had a similar aspect and size to the same formulation with the coating (Figure 1 O, OD-AC; O, OD-BC), suggesting the very low thickness of the silica coating (2.75 ± 0.49 nm). This ultrathin silica coating is beneficial to the energy transfer between UCNPs [35]. The mid-term storage (2 years) of UCNPs under controlled conditions had no effect on particle morphology (Figure 1 O, OD-BC just after synthesis vs. 2 years after synthesis, and N, OD-BC just after synthesis vs. 2 years after synthesis). Regarding UCNPs sizes, the major population of the O, OD-BC formulation obtained just after synthesis was lost after 2-years of mid-term storage, in contrast to the N, OD-BC formulation, where the UCNP size was preserved after mid-term storage. Therefore, results suggest that the UCNPs obtained from the lanthanide nitrates are more stable in terms of size than the ones obtained from the lanthanide oxides. This morphological stability is adequate and essential to satisfy the requirements of biological applications [35].

Aggregates were detected for some UCNPs formulations (Figure 1 N, OD-AC; O, OD-AC; O, OD and N, SCD-AC) with a decrease in their formation after mid-term storage (O, OD-BC just after synthesis vs. O, OD-BC 2 years after synthesis and N, OD-BC just after synthesis vs. N, OD-BC 2 years after synthesis). UCNPs tended to aggregate after annealing at higher temperatures and also due to the carbonization of the capping reagent EDTA after annealing, thus reducing UCNPs hydrophilicity, in agreement with observations by Wang and coworkers [21]. The aggregation may also be a consequence of the interparticle chemical bonding between the silica shells of the UCNPs [31]. It was previously described that there is an optimal size for efficient uptake of each type of nanomaterials into cells [36]. UCNPs of a very low size (<45 nm) were reported with the same sensitizers (Yb^3+^ and Er^3+^) [20,37,38], which could be easily internalized by cells and induce high toxicity afterwards. In contrast, this particle size range just represents a very minor population in all UCNPs formulations (Figure 1) and adequate sizes for biomedical applications were obtained with this novel co-precipitation approach herein developed. Furthermore, an increase in the size of the nanocrystals obtained with the same trivalent lanthanide ions was reported to increase the luminescence emission [39].

**Figure 2:**
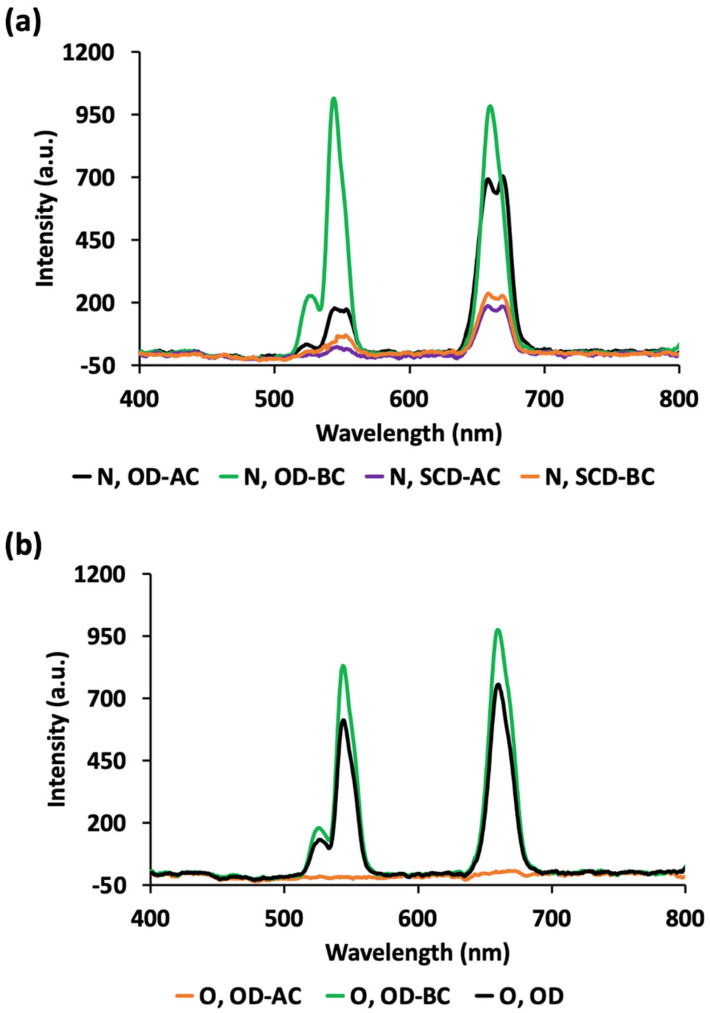
UC spectra for the (**a**) lanthanide-nitrates-based UCNPs and (**b**) lanthanide-oxides-based UCNPs in the solid state, collected with λ_exc_ = 980 nm. For notation of the formulations, please refer to Table 1.

All the synthesized UCNPs emitted visible radiation when excited at 980 nm, showing three different bands at 520, 540 and 657 nm in the UC luminescence spectra (Figure 2a,b), except for the oxide-derivative O, OD-AC formulation where no meaningful UC emission was detected (Figure 2b). The lower efficiency of the UC process for the O, OD-AC formulation can be attributed to the type of host matrix phase (cubic α-NaYF_4_ or hexagonal β-NaYF_4_) [18,19,21], which will be discussed later. The peaks at 520, 540 and 657 nm correspond to ^2^H_11/2_ → ^4^I_15/2_, ^4^S_3/2_ → ^4^I_15/2_, ^4^F_9/2_ → ^4^I_15/2_ optical radiative transitions of Er^3+^ ions, respectively, as previously reported for UCNPs composed of the same trivalent lanthanide ions [39,40] and in a study of Er^3+^ and Yb^3+^ UC photoluminescence on films [41]. It must be noted that an enhancement of the photoluminescence was observed when structures where co-doped with both ions, with a photoluminescence intensity more than one order-of-magnitude higher with respect to materials doped only with Er^3+^ [41].

Some formulations (N, OD-AC; N, SCD-AC and N, SCD-BC) exhibited structured green and red bands maybe due to the saturation of the detector and not to the emission from different excited states [42]. Similar structured bands were also reported for different NaYF_4_:Yb^3+^, Er^3^ formulations [38,39,43]. In the comparative UC spectra performed under constant experimental conditions (laser intensity), the N, OD-BC and O, OD-BC formulations exhibited a higher fluorescence intensity in each peak, achieving the highest values for N, OD-BC. It has been recently published that with a very low Er^3+^ concentration (close to 1%) UC photoluminescence was not detected, so the conversion of NIR into visible light comes from the interaction between neighboring Er^3+^ ions [44]. This reaction was associated with the energy transfer from one excited Er^3+^ to another Er^3+^ and with the upgrade of the latter Er^3+^ into a higher-energy level. In this cooperative UC, Er^3+^ luminescence comes from two centers, one related to the Er^3+^ UC luminescence (520 and 540 nm peaks) and the other one related to the Er^3+^-Yb^3+^ ion-ion interaction and its UC luminescence (657 nm peak). It is also important to note that the green band/red band UC emission intensity ratio could be related to different “excitation environments” in terms of ion size, ionic dependence of site distortions and ion concentration due to a concentration-quenching effect between Er^3+^ and Yb^3+^ ions [40,44].

**Figure 3:**
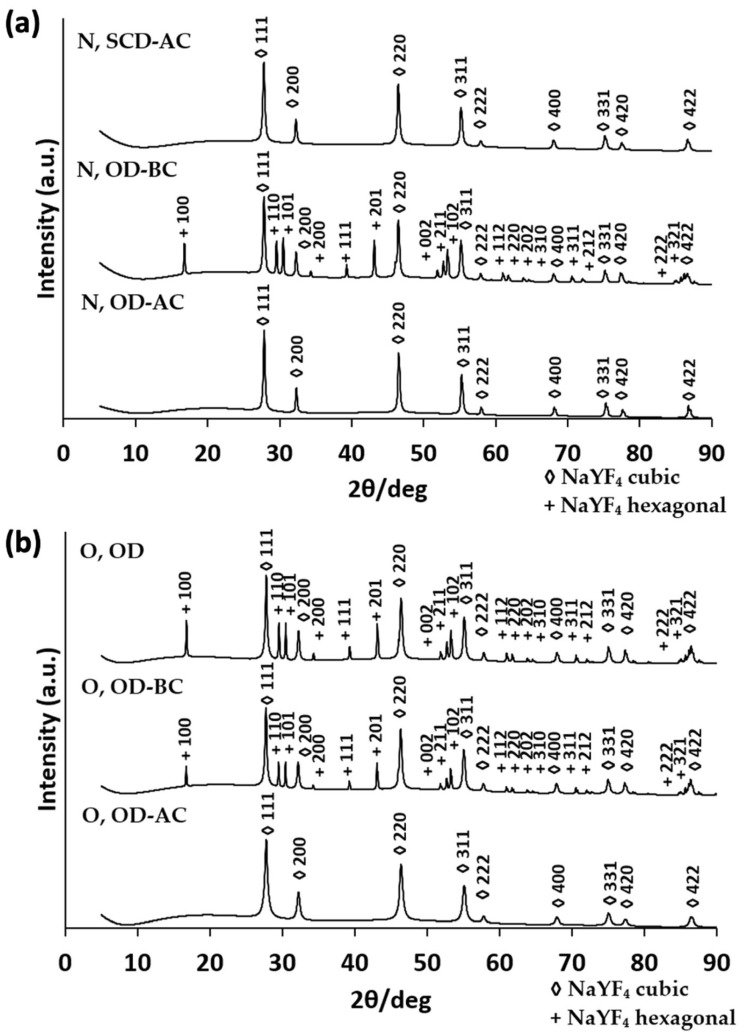
XRD diffractograms of (**a**) lanthanide nitrates-based UCNPs and (**b**) lanthanide oxides-based UCNPs. For notation of the formulations, please refer to Table 1.

The crystalline nature of UCNPs was confirmed by the corresponding XRD pattern (Figure 3a,b), with similar diffractograms to those previously reported for UCNPs with the same sensitizer–activator system (Yb^3+^-Er^3+^) [45]. The UC process is improved with a highly crystalline host lattice holding a low-energy environment that enables the energy transfer from the sensitizer to the activator. The essential requirements for an efficient energy transfer are the distance between the sensitizer and the activator, the spectral overlap and the achievement of a high absorbance by the activator at the emission wavelength of the sensitizer [46]. Both types of crystalloid phases, cubic and hexagonal, exist at the ambient pressure being the cubic phase the dominant one, i.e., the thermodynamically metastable. However, the green emission of the crystalline hexagonal phase is 10 times stronger than that of the crystalline cubic phase and the overall emission is ca. 5-fold higher in the thermodynamically stable β-NaYF_4_:Yb^3+^, Er^3+^. Nevertheless, there is a high-energy barrier between α- and β-phases, so the phase transition needs temperatures higher than 280 °C [37]. It has also been reported that both phases coexist from 290 to 300 °C and that very long reaction times are needed for the complete α → β transition at 300 °C, so 400 °C was herein selected as the reaction temperature. Consistently, the formation of β-phased UCNPs did not involve a direct phase transformation process but a dissolution/recrystallization process. As expected, the fluorescence intensity increased according to the UCNP conversion from the host matrix cubic to the hexagonal phase after annealing at 400 °C. Nevertheless, for N, SCD-AC, N, OD-AC and O, OD-AC formulations, no peaks related to the hexagonal phase were observed (Figure 3a,b). The hexagonal phase peaks were detected for N, OD-BC, O, OD-BC and O, OD formulations, thus suggesting that the oven drying is a more appropriate method for the synthesis of UCNPs with respect to the SCD. Finally, the highest fluorescence intensity appeared when drying and annealing steps were performed before the silica coating step, so the best formulations in terms of fluorescence intensity (O, OD; O, OD-BC; N, OD-BC) were selected for further EDS characterization. The full accomplishment of the crystalline hexagonal phase under certain experimental conditions unveils the phase-controlling ability of the co-precipitation method herein employed.

**Figure 4:**
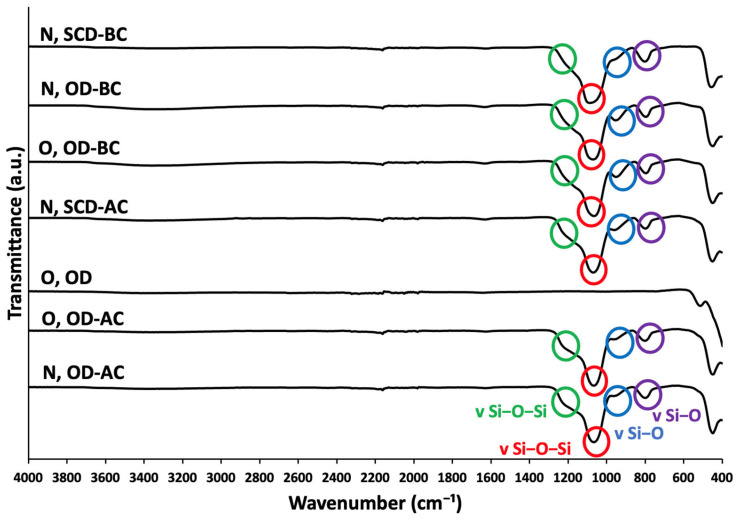
FTIR spectra of different formulations of UCNPs. For notation of the formulations, please refer to Table 1.

FTIR spectroscopy was used to trace the coating step with silica of the different UCNPs formulations. The presence of the silica was confirmed in all formulations where the coating step was performed by the detection of two broad bands at 1080 cm^−1^ (Figure 4, red circles) and 1175 cm^−1^ (green circles) corresponding to vSi-O-Si vibrations. Moreover, bands at 970 cm^−1^ (blue circles) and 800 cm^−1^ (purple circles), corresponding to a vSi-O vibration of the ≡Si-O-Si≡ bond, were detected and associated with the silica functionalization [47,48]. FTIR spectra of all samples are almost completely smooth in the 1400–4000 cm^−1^ region, without exhibiting characteristic peaks that could indicate the presence of functional groups not related to silica. Results confirmed that the functionalization of UCNPs with silica was successfully achieved regardless of the drying procedure used (SCD or oven drying) or the moment when the coating was performed (before or after the drying step).

**Figure 5:**
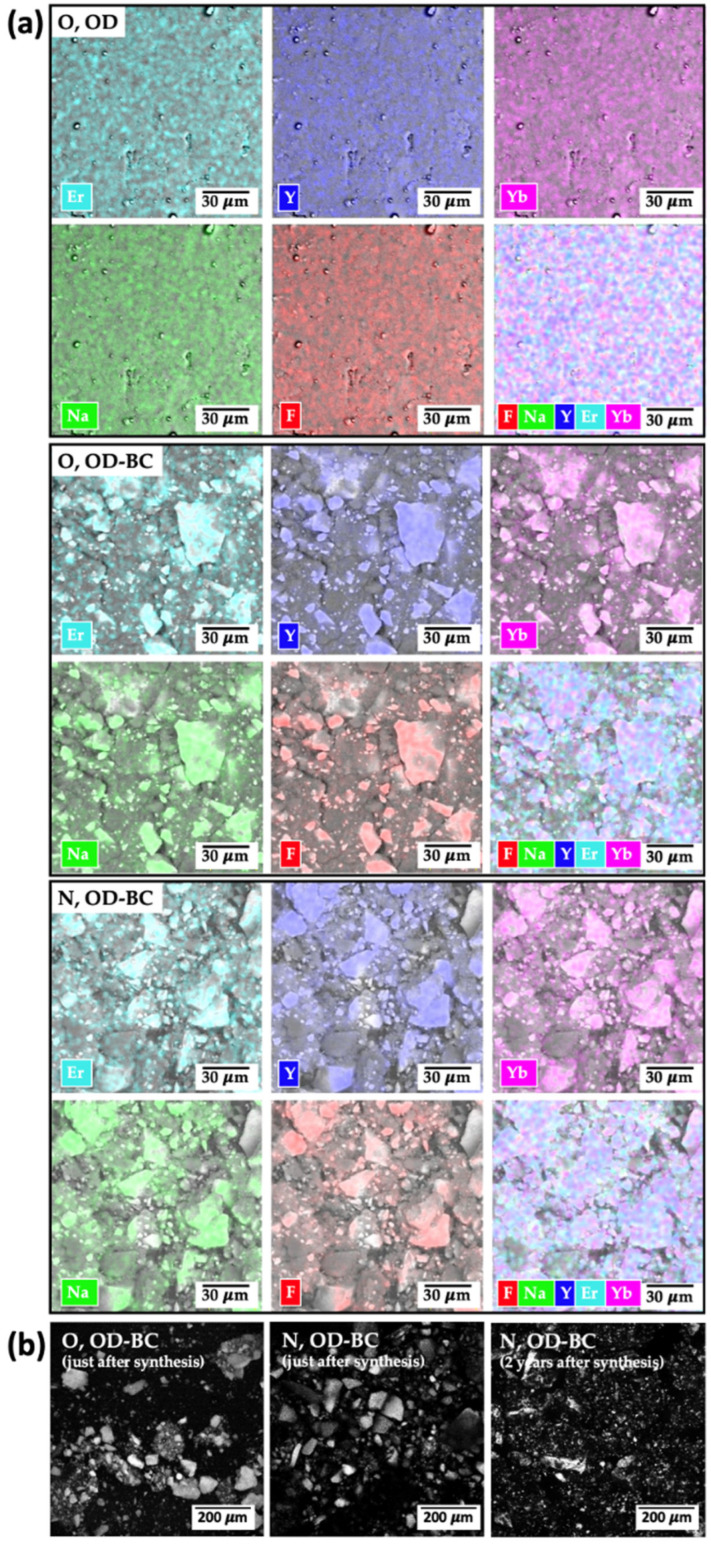
(**a**) EDS mapping and (**b**) confocal microscopy images of different UCNPs formulations. For notation of the formulations, please refer to Table 1.

The elemental mapping obtained by the EDS technique (Figure 5a) confirmed the presence of all the employed chemical elements, including the peaks related to ions present at low concentrations (Na and F). UCNPs showed the peaks related to the host material constituents and lanthanides homogeneously distributed throughout the analyzed sample, thus confirming their expected composition and that they were congruent with the obtained UC fluorescence results. EDS results also unveiled the decoration of UCNPs with similar intensity for the elements from the host matrix (Na, F) and for the rare-earth elements (Y, Yb, Er). This elemental mapping suggests their simultaneous and homogeneous distribution throughout the sample, which is attributed to the successful connection between the rare-earth elements and the host matrix. Similar elemental mapping was previously found for UCNPs doped with the same rare-earth elements [35]. The background material with lower fluorescence is mainly silica. The more homogeneous formulations in terms of fluorescence are the lanthanide nitrate-based UCNPs. This is a very beneficial result since the nitrate-derived UCNPs are much easier to synthesize than their oxide-derived counterparts and only involve non-hazardous steps. Since high crystallinity and the presence of chemical elements were confirmed for all formulations studied, the best formulation in terms of fluorescence intensity (i.e., the formulation with the highest intensity in each peak, N, OD-BC) was selected for biological characterization (cf. Section 2.2). This formulation also exhibited physicochemical stability in terms of particle shape and fluorescence properties after a 2-year mid-term storage.

Confocal microscopy is the main approach used for localizing UCNPs by in vitro cell-based tests and by in vivo bioimaging experiments. For this reason, the best formulations of UCNPs were also analyzed by this technique (Figure 5b), with a good fluorescence signal obtained just after synthesis (for both formulations studied) and after 2-year storage, thus endorsing the possibility of their mid-to-long-term storage without detrimental in fluorescence properties. Long-term photostability was previously studied for UCNPs [32] with good results in terms of UC spectra but after shorter time periods (5 months) than those herein evaluated.

### 2.2. Biocompatibility and Hemocompatibility Evaluation

**Figure 6:**
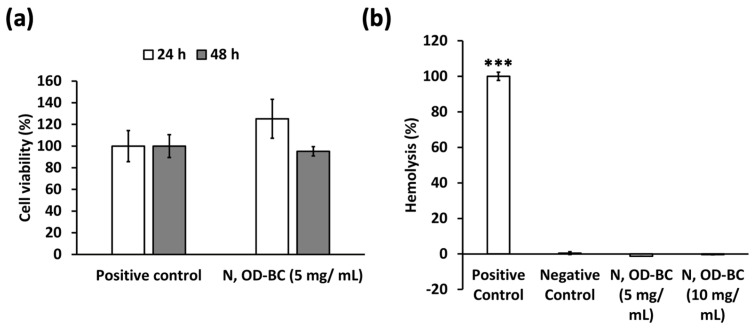
(**a**) Cell viability of BALB/c3T3 cells in contact with N, OD-BC UCNPs at 5 mg/mL determined by WST-1 test. Positive control: BALB/c3T3 cells. No statistically significant differences among groups were detected (post hoc Tukey HSD multiple comparison-test; *p* < 0.05). (**b**) Hemolytic activity (expressed in %) for N, OD-BC UCNPs (5 and 10 mg/mL). Positive and negative controls were 4% (*v*/*v*) Triton X-100 solution and PBS pH 7.4 solution, respectively. Statistically significant differences among groups were represented as *** (post hoc Tukey HSD multiple comparison test; *p* < 0.001). For notation of the formulation, please refer to Table 1.

The biocompatibility of UCNPs was confirmed by the high viability of mouse embryo fibroblasts in the presence of the N, OD-BC UCNP formulation for 24 and 48 h, without significant differences with respect to the positive controls (Figure 6a). These results are better than those previously reported for silica-coated NaYF_4_:Yb^3+^, Er^3+^ UCNPs with a low size (21 ± 5 nm) after contact with bone marrow-derived stem cells (79.5% after 24 h and 66.8% after 48 h) or skeletal myoblasts (87.8% after 24 h and 68.2% after 48 h) at 100 μg/mL [49]. Concentrations (0–5 mg/mL) close to those herein reported (5 mg/mL) were previously tested for KGdF_4_:Yb^3+^, Er^3+^ UCNPs with cell viabilities higher than 60% after 20 h [50]. Moreover, lower concentrations of UCNPs (100 μg/mL) showed lower levels of biocompatibility than those herein reported (close to 75% of cell viability) and were reported as safe to be used in subcellular imaging applications [51]. A reduction in the viability after 72 h of rat glioma cells and of rat mesenchymal stem cells was observed for lower concentrations than those herein studied (1000 μg/mL) of polymer-coated UCNPs composed of the same trivalent lanthanide ions [39]. Furthermore, the relevance of the protective coating to the safety of NPs was assessed by evaluating the cell viability in NaYF_4_:Yb^3+^, Er^3^UCNPs with different polymeric coatings [38]. Very low cell viabilities were obtained with neat UCNPs while the different polymer coatings studied increased rat mesenchymal stem cell viabilities, evaluated in the concentration range 0–1000 µg/ mL. It is important to note that much lower concentrations than those herein tested (100 µg/mL) showed a bright fluorescence signal on HeLa cells, with a high cell viability (90%) after 24 h [32]. These findings emphasized the safety of use of the herein synthesized N, OD-BC UCNPs.

No hemolysis was observed on human red blood cells after contact with N, OD-BC UCNPs (Figure 6b), with no significant differences with respect to the negative controls for the two studied concentrations (5 and 10 mg/mL). No influence of UCNPs on rat red blood cells was previously reported for NaYF_4_:Yb^3+^, Er^3+^,Gd^3+^ UCNPs at lower UCNPs concentrations (25–500 μg/mL) [35]. Excellent human blood compatibility was also found for NaGdF_4_:Yb^3+^, Er^3+^ UCNPs at a Gd content of 500 ppm [45]. These results suggest the absence of a negative impact of the N, OD-BC UCNPs, even at very high concentrations (10 mg/mL), on human red blood cells. Therefore, silica coating by sol-gel Stöber process seems to create a structurally stable nanocomplex in biological fluids that effectively protect the lanthanide core from inducing any cytotoxic event.

## 3. Conclusions

NaYF_4_:Yb^3+^, Er^3+^ core-shell nanocomplexes with a highly photostable luminescent core and a non-porous silica gel shell were successfully fabricated by a facile and environmentally friendly combination of co-precipitation (core) and sol-gel (shell) approaches. The synthetic strategy was able to control the morphology and crystalline phase of the UCNPs as well as their biocompatibility. The size and ζ-potential values achieved suggest the absence of induction of negative cell interactions. Similar morphology and luminescence of silica gel-coated UCNPs were observed after 2-year mid-term storage, thus indicating their preliminary physicochemical stability. An optimum formulation with the highest values of fluorescence intensity and coherent presence of all elements in the UCNPs was selected. Excellent bio- and hemocompatibility were obtained for these UCNPs, after assays with murine fibroblast and human blood, respectively. The protective silica coating synthesized by the sol-gel Stöber process seems to preserve over time the lanthanide core UC emission while promoting the bio- and hemocompatibility of the nanosystem. Consistently, these UCNPs represent a promising alternative to be tested as in vivo biolabels, as the concentration range could be increased until 5 mg/mL to get a detectable signal without losing bio- or hemocompatibility. These results encourage further research into potential applications of the herein synthesized silica gel-coated UCNPs as biomarkers for biological detections, in cellular and animal imaging systems by their incorporation into biomedical devices for further evaluation of these UCNPs as in vivo bioimaging probes.

## 4. Materials and Methods

### 4.1. Materials

Ytterbium (III) oxide (Yb_2_O_3_, 99.9% purity), erbium (III) oxide (Er_2_O_3_, 99.9%), ytterbium (III) nitrate pentahydrate (Yb(NO_3_)_3_·5H_2_O, 99.9%), yttrium(III) nitrate hexahydrate (Y(NO_3_)_3_·6H_2_O, 99.8%), sodium fluoride (NaF, >99%) and ethylenediaminetetraacetic acid (EDTA) were provided by Sigma Aldrich (Merck Group, Darmstadt, Germany). Yttrium (III) oxide (Y_2_O_3_, 99.99%), erbium (III) nitrate pentahydrate (Er(NO_3_)_3_·5H_2_O, 99.9%), and tetraethyl orthosilicate (TEOS, Si(OC_2_H_5_)_4_, 98%), were obtained from Acros Organics (Thermo-Fisher Scientific, Geel, Belgium). Hydrochloric acid (HCl, 37%) was supplied by Fisher (Madrid, Spain) and ammonium hydroxide (25% NH_3_ in H_2_O) by Fluka Analytical (Honeywell International, Charlotte, NC, USA). CO_2_ (purity > 99.9%) was obtained from Nippon Gases (Madrid, Spain) and absolute ethanol (EtOH) from VWR (Radnor, PA, USA). All reagents were used as purchased and deionized water was employed in all the procedures.

### 4.2. Synthesis of UCNPs by Co-Precipitation and Sol-Gel Methods

0.2 M stock solutions of Y_2_O_3_, Yb_2_O_3_, and Er_2_O_3_ solutions were prepared and adjusted at pH 2 by using hydrochloric acid to dissolve the oxide precursors and liberate the metal ions to the solution [52]. A total of 16 mL of 0.2 M Y_2_O_3_, 3.4 mL of 0.2 M Yb_2_O_3_ and 0.6 mL of 0.2 M Er_2_O_3_ was mixed with 20 mL of 0.2 M EDTA. Alternatively, another solution was prepared by mixing 16 mL of 0.2 M Y(NO_3_)_3_, 3.4 mL of 0.2 M Yb(NO_3_)_3_, 0.6 mL of 0.2 M Er(NO_3_)_3_ and 20 mL of 0.2 M EDTA. EDTA was firstly employed in the synthesis of UCNPs by Yi and coworkers [52]. It is employed as a stabilizing and chelating agent able to minimize UCNPs aggregation [53]. EDTA has been described as an efficient complexing agent for trivalent lanthanide ions because it improves the dispersibility of crystals. Complexes formed are adsorbed on the surface of NPs, limiting the growth in size of the resulting UCNPs. In fact, smaller NPs size is generally achieved by increasing the concentration of different chelators [54]. Moreover, EDTA is used to prevent precipitation before proceeding with the synthesis procedure [55].

Rare earth solution was injected into 0.05 mol of NaF dissolved in 60 mL of water and the mixture was stirred for 1 h at RT. Obtained dispersions were centrifuged and washed three times with water and once with ethanol. NPs were dried in an oven at 60 °C or, alternatively, under SCD with EtOH. For the SCD, NPs dispersed in EtOH were placed inside an autoclave filled with EtOH, sealed, and flushed with gaseous N_2_. Pressure was maintained at 80 bar until reaching 260 °C with a heating rate of 80 °C/h. The reactor valve outlet was opened for EtOH release under supercritical conditions, thus dropping the pressure gradually. The system was cooled down to RT before opening the autoclave. NPs annealing was carried out under a N_2_ atmosphere, for 5 h and 400 °C with a heating rate of 20 °C/min. NPs were cooled down until RT under the same atmosphere.

To obtain the silica shell, 30 mL of NPs dispersed in ethanol (3 mg/mL) were placed in a bath at 0 °C under stirring [47]. TEOS (0.2 mL) was firstly added and, 5 min later, ammonium hydroxide (3 mL) was then incorporated. The reaction took place for 2 h, under stirring and at 0 °C. The thus obtained NPs were washed three times with EtOH.

**Figure 7:**
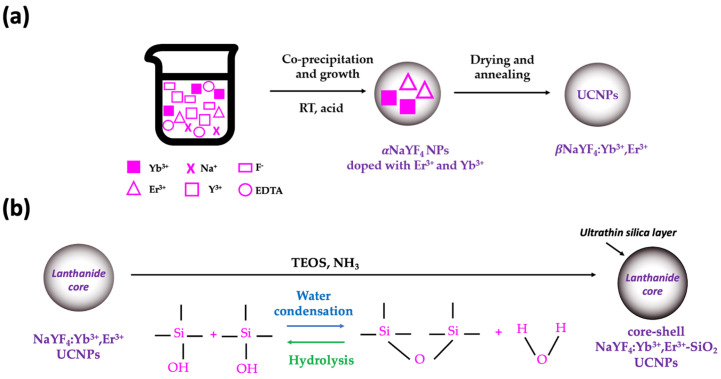
Experimental procedure used to obtain silica gel-coated UCNPs: (**a**) Synthesis of UCNPs by co-precipitation, and (**b**) production of core–shell NaYF_4_:Yb^3+^, Er^3+^-SiO_2_ UCNPs by sol–gel Stöber method. The simplified chemical reactions of the sol–gel method, hydrolysis, and condensation of TEOS, are also included. Silica gel coating method was performed either before or after the drying step.

Synthesis of the lanthanide core-shell UCNPs was thus performed according to the scheme depicted in Figure 7a,b.

### 4.3. Physicochemical Evaluation of UCNPs

ζ-potential measurements of UCNPs were performed with a ζ-potential analyzer (Malvern Panalytical, Malvern, UK). The morphology of UCNPs was evaluated by transmission electron microscopy (TEM, JEOL JEM-2010, Tokyo, Japan) operating at 200 kV. Confocal microscopy (Leica TCS-SP2 spectral confocal microscope, Leica Microsystems Heidelberg GmbH, Mannheim, Germany) was also used to qualitatively evaluate the fluorescence emission of the UCNPs powder. A 2-year physicochemical stability of UCNPs was evaluated for the most promising formulations in terms of luminescent properties by comparative TEM and confocal microscopy analysis, performed just after synthesis and after a 2-year mid-term storage of the UCNPs at RT, protected from light and stored in closed glass vials during this time.

To obtain the luminescence measurements and verify the UC process in the nanocomplexes, the UC emission spectra of the UCNPs in a 2 mm cuvette were collected in a sample holder with a ~45° angle with respect to the excitation. The excitation source consisted of a Spectra Physics Solstice-Ace laser coupled to a TOPAS Prime Amplifier, with an excitation wavelength of 980 nm (λ_exc_ = 980 nm) while the emission was recorded using an AvaSpec-ULS-TEC Avantes Senseline Fiber Optic Spectrometer System. A Newport 10SWF-800-B (BG059) short-bandpass filter was used in the emission optical path to cut the laser-scattered light. The UC spectra were collected in the 450–725 nm emission range.

Crystalline phases of the nanocomplexes were identified by X-ray diffraction (XRD) using a Bruker 8D advance diffractometer (Billerica, MA, USA). Diffractograms were obtained using Cu Kα radiation (λ = 0.154184 nm) in the 5–120° (2θ) range. The step was set at 0.03°, the recording time at 7 s per step, and the applied voltage and current at 40 kV and 40 mA, respectively. Energy-dispersive X-ray spectroscopy (EDS) was performed with a TESCAN VEGA3 SBH (Brno, Czech Republic) microscope with an EDS detector Burker XFlagh 410 M (Billerica, MA, USA) operating at 20 kV. Attenuated total reflectance/Fourier-transform infrared spectroscopy (ATR/FT-IR) was obtained with a Gladi-ATR accessory. A diamond crystal (Pike, Madison, WI, USA) was employed to analyze the chemical structure of the nanocomplexes in the mid-IR spectrum (400–4000 cm^−1^) with 32 scans and a resolution of 2 cm^−1^.

### 4.4. Viability of Mouse Embryo Fibroblasts

Cytocompatibility of the most promising UCNPs formulation (N, OD-BC) was evaluated by assessing the degradation of WST-1 into formazan, which directly correlates with the number of cells that are metabolically active. BALB/c3T3 cells (6500 cells/cm^2^) were seeded in 24-well plates with 1000 µL of DMEM supplemented with 15% fetal bovine serum, penicillin 100 U/mL and streptomycin 100 g/mL. After incubation at 37 °C in a humidified atmosphere enriched with 5% CO_2_ for 24 h, UCNPs (N, OD-BC) in powder form (5 mg) were UV-sterilized for 30 min and placed in a culture insert in contact with cells by triplicate. The previous literature does not evaluate concentrations above 5 mg/mL for bioimaging applications, including in vivo experiments, since lower amounts must provide a detectable fluorescence signal without compromising biocompatibility [21,28,32]. Cells alone were also tested under the same conditions as the positive controls. After 24 and 48 h of culture, inserts were removed, 25 µL of WST-1 was pipetted and plates were incubated for 2 h. Some 110 µL were then pipetted to a 96-well plate and absorbance was measured at λ = 450 nm (Infinite^®^ M200, Tecan Group Ltd., Männedorf, Switzerland).

### 4.5. Hemolytic Activity of Human Red Blood Cells

The hemolytic activity of 5 and 10 mg of the most promising formulation of UCNPs (N, OD-BC) was tested by contact with human blood. Fresh human whole blood was obtained from the Galician Transfusion Center (Spain) in accordance with the Declaration of Helsinki. Firstly, blood was diluted to 3% (*v*/*v*) in 0.9% (*w*/*v*) NaCl. Then, 1000 µL of the diluted blood was poured into conical tubes containing different amounts of UCNPs (5 or 10 mg). Furthermore, 900 μL of the diluted blood was mixed with 100 μL of 4% (*v*/*v*) Triton X-100 (positive control) or 100 μL of 0.9% (*w*/*v*) PBS pH 7.4 (negative control). Samples were tested by triplicate and incubated at 37 °C and 100 rpm in an orbital shaker for 60 min. After centrifugation at 10.000g for 10 min (Sigma 2-16P, Sigma Laboratory Centrifuges, Osterode am Harz, Germany), 150 μL of the supernatant were pipetted into a 96-well plate to measure the hemoglobin absorbance at λ = 540 nm (FLUOStar Optima, BMG Labtech, Ortenberg, Germany). Hemolysis was determined by Equation (1):

Hemolysis (%) = (Abs_s_ − Abs_n_)/(Abs_p_ − Abs_n_) × 100 (1)

where Abs_s_ is the absorbance of the samples, Abs_n_ is the absorbance of the negative control (0% of hemolysis), and Abs_p_ is the absorbance of the positive control (100% of hemolysis).

### 4.6. Statistical Analysis

Results of in vitro assays (*n* = 3) were reported as the mean value ± standard deviation and post hoc Tukey HSD multiple comparison tests were performed to assess the statistical significance of the differences between groups and regarding controls. Values of *p* < 0.05 were considered statistically significant.

## Figures and Tables

**Table 1:** Different UCNPs formulations studied.

Formulation	Lanthanide Precursors	Drying Method	Drying Moment
O, OD-AC	Lanthanide oxides	Oven drying	After silica coating
O, OD-BC	Lanthanide oxides	Oven drying	Before silica coating
O, OD	Lanthanide oxides	Oven drying	-
N, OD-AC	Lanthanide nitrates	Oven drying	After silica coating
N, OD-BC	Lanthanide nitrates	Oven drying	Before silica coating
N, SCD-AC	Lanthanide nitrates	SCD	After silica coating
N, SCD-BC	Lanthanide nitrates	SCD	Before silica coating

**Table 2:** ζ-potential measurements of different UCNPs formulations. For notation of the formulations, please refer to Table 1.

UCNPs Formulation	ζ-Potential (mV)
O, OD-AC	−7.28 ± 1.99
O, OD-BC	−17.72 ± 0.12
O, OD	−6.39 ± 2.03
N, OD-AC	−9.64 ± 1.03
N, OD-BC	−7.07 ± 1.32
N, SCD-AC	−9.04 ± 1.09
N, SCD-BC	−8.78 ± 0.52

## Data Availability

The data presented in this study are openly available in article.

## References

[1] Lahoti H.S., Jogdand S.D. (2022). Bioimaging: Evolution, Significance, and Deficit. Cureus.

[2] Wang C., Li X., Zhang F. (2016). Bioapplications and Biotechnologies of Upconversion Nanoparticle-Based Nanosensors. Analyst.

[3] Hu Y., Wu B., Jin Q., Wang X., Li Y., Sun Y., Huo J., Zhao X. (2016). Facile Synthesis of 5 Nm NaYF4:Yb/Er Nanoparticles for Targeted Upconversion Imaging of Cancer Cells. Talanta.

[4] Gerelkhuu Z., Lee Y.-I., Yoon T.H. (2022). Upconversion Nanomaterials in Bioimaging and Biosensor Applications and Their Biological Response. Nanomaterials.

[5] Malhotra K., Hrovat D., Kumar B., Qu G., Houten J.V., Ahmed R., Piunno P.A.E., Gunning P.T., Krull U.J. (2023). Lanthanide-Doped Upconversion Nanoparticles: Exploring A Treasure Trove of NIR-Mediated Emerging Applications. ACS Appl. Mater. Interfaces.

[6] Mahata M.K., De R., Lee K.T. (2021). Near-Infrared-Triggered Upconverting Nanoparticles for Biomedicine Applications. Biomedicines.

[7] Vorotnikov Y.A., Vorotnikova N.A., Shestopalov M.A. (2023). Silica-Based Materials Containing Inorganic Red/NIR Emitters and Their Application in Biomedicine. Materials.

[8] Le X.T., Youn Y.S. (2020). Emerging NIR Light-Responsive Delivery Systems Based on Lanthanide-Doped Upconverting Nanoparticles. Arch. Pharm. Res..

[9] Tsai S.-R., Hamblin M.R. (2017). Biological Effects and Medical Applications of Infrared Radiation. J. Photochem. Photobiol. B Biol..

[10] Gnach A., Lipinski T., Bednarkiewicz A., Rybka J., Capobianco J.A. (2015). Upconverting Nanoparticles: Assessing the Toxicity. Chem. Soc. Rev..

[11] Tanaka Y., Tsunemi Y., Kawashima M., Nishida H. (2013). The Impact of Near-Infrared in Plastic Surgery. Plast. Surg. Int. J..

[12] Zhu X., Zhang J., Liu J., Zhang Y. (2019). Recent Progress of Rare-Earth Doped Upconversion Nanoparticles: Synthesis, Optimization, and Applications. Adv. Sci..

[13] Yi Z., Luo Z., Qin X., Chen Q., Liu X. (2020). Lanthanide-Activated Nanoparticles: A Toolbox for Bioimaging, Therapeutics, and Neuromodulation. Acc. Chem. Res..

[14] Liu Q., Chen M., Sun Y., Chen G., Yang T., Gao Y., Zhang X., Li F. (2011). Multifunctional Rare-Earth Self-Assembled Nanosystem for Tri-Modal Upconversion Luminescence /Fluorescence /Positron Emission Tomography Imaging. Biomaterials.

[15] Cheng L., Yang K., Shao M., Lu X., Liu Z. (2011). In Vivo Pharmacokinetics, Long-Term Biodistribution and Toxicology Study of Functionalized Upconversion Nanoparticles in Mice. Nanomedicine.

[16] Generalova A.N., Chichkov B.N., Khaydukov E.V. (2017). Multicomponent Nanocrystals with Anti-Stokes Luminescence as Contrast Agents for Modern Imaging Techniques. Adv. Colloid Interface Sci..

[17] Min Y., Li J., Liu F., Padmanabhan P., Yeow E., Xing B. (2014). Recent Advance of Biological Molecular Imaging Based on Lanthanide-Doped Upconversion-Luminescent Nanomaterials. Nanomaterials.

[18] Zhao J., Lu Z., Yin Y., McRae C., Piper J.A., Dawes J.M., Jin D., Goldys E.M. (2013). Upconversion Luminescence with Tunable Lifetime in NaYF_4_: Yb,Er Nanocrystals: Role of Nanocrystal Size. Nanoscale.

[19] Lingeshwar Reddy K., Balaji R., Kumar A., Krishnan V. (2018). Lanthanide Doped Near Infrared Active Upconversion Nanophosphors: Fundamental Concepts, Synthesis Strategies, and Technological Applications. Small.

[20] Homann C., Krukewitt L., Frenzel F., Grauel B., Würth C., Resch-Genger U., Haase M. (2018). NaYF_4_: Yb,Er/NaYF_4_ Core/Shell Nanocrystals with High Upconversion Luminescence Quantum Yield. Angew. Chem. Int. Ed..

[21] Wang M., Abbineni G., Clevenger A., Mao C., Xu S. (2011). Upconversion Nanoparticles: Synthesis, Surface Modification and Biological Applications. Nanomed. Nanotechnol. Biol. Med..

[22] Yang L., Shao B., Zhang X., Cheng Q., Lin T., Liu E. (2016). Multifunctional Upconversion Nanoparticles for Targeted Dual-Modal Imaging in Rat Glioma Xenograft. J. Biomater. Appl..

[23] Alkahtani M., Alsofyani N., Alfahd A., Almuqhim A.A., Almughem F.A., Alshehri A.A., Qasem H., Hemmer P.R. (2021). Engineering Red-Enhanced and Biocompatible Upconversion Nanoparticles. Nanomaterials.

[24] Rafique R., Baek S.H., Park C.Y., Chang S.-J., Gul A.R., Ha S., Nguyen T.P., Oh H., Ham S., Arshad M. (2018). Morphological Evolution of Upconversion Nanoparticles and Their Biomedical Signal Generation. Sci. Rep..

[25] Zhou R., Ma T., Qiu B., Li X. (2017). Controlled Synthesis of β-NaYF 4:Yb, Er Microphosphors and Upconversion Luminescence Property. Mater. Chem. Phys..

[26] Xu D., Li C., Li W., Lin B., Lv R. (2023). Recent Advances in Lanthanide-Doped up-Conversion Probes for Theranostics. Front. Chem..

[27] Yan C., Zhao H., Perepichka D.F., Rosei F. (2016). Lanthanide Ion Doped Upconverting Nanoparticles: Synthesis, Structure and Properties. Small.

[28] Sun Y., Peng J., Feng W., Li F. (2013). Upconversion Nanophosphors Nalu_4_: Yb,Tm for Lymphatic Imaging In Vivo by Real-Time Upconversion Luminescence Imaging under Ambient Light and High-Resolution X-Ray CT. Theranostics.

[29] Stöber W., Fink A., Bohn E. (1968). Controlled Growth of Monodisperse Silica Spheres in the Micron Size Range. J. Colloid Interface Sci..

[30] Gonçalves M.C. (2018). Sol-Gel Silica Nanoparticles in Medicine: A Natural Choice. Design, Synthesis and Products. Molecules.

[31] Muhr V., Wilhelm S., Hirsch T., Wolfbeis O.S. (2014). Upconversion Nanoparticles: From Hydrophobic to Hydrophilic Surfaces. Acc. Chem. Res..

[32] Liu F., Zhao Q., You H., Wang Z. (2013). Synthesis of Stable Carboxy-Terminated NaYF_4_: Yb^3+^, Er^3+^@SiO_2_ Nanoparticles with Ultrathin Shell for Biolabeling Applications. Nanoscale.

[33] Guryev E.L., Smyshlyaeva A.S., Shilyagina N.Y., Sokolova E.A., Shanwar S., Kostyuk A.B., Lyubeshkin A.V., Schulga A.A., Konovalova E.V., Lin Q. (2020). UCNP-Based Photoluminescent Nanomedicines for Targeted Imaging and Theranostics of Cancer. Molecules.

[34] Jin J., Gu Y.-J., Man C.W.-Y., Cheng J., Xu Z., Zhang Y., Wang H., Lee V.H.-Y., Cheng S.H., Wong W.-T. (2011). Polymer-Coated NaYF_4_: Yb^3+^, Er^3+^ Upconversion Nanoparticles for Charge-Dependent Cellular Imaging. ACS Nano.

[35] Chen J., Zhang D., Zou Y., Wang Z., Hao M., Zheng M., Xue X., Pan X., Lu Y., Wang J. (2018). Developing a pH-Sensitive Al(OH)_3_ Layer-Mediated UCNP@Al(OH)_3_/Au Nanohybrid for Photothermal Therapy and Fluorescence Imaging in vivo. J. Mater. Chem. B.

[36] Verma A., Stellacci F. (2010). Effect of Surface Properties on Nanoparticle—Cell Interactions. Small.

[37] Li D., Shao Q., Dong Y., Jiang J. (2014). Phase-, Shape- and Size-Controlled Synthesis of NaYF_4_:Yb^3+^, Er^3+^ Nanoparticles Using Rare-Earth Acetate Precursors. J. Rare Earths.

[38] Nahorniak M., Patsula V., Mareková D., Matouš P., Shapoval O., Oleksa V., Vosmanská M., Machová Urdzíková L., Jendelová P., Herynek V. (2023). Chemical and Colloidal Stability of Polymer-Coated NaYF_4_:Yb, Er Nanoparticles in Aqueous Media and Viability of Cells: The Effect of a Protective Coating. Int. J. Mol. Sci..

[39] Patsula V., Mareková D., Jendelová P., Nahorniak M., Shapoval O., Matouš P., Oleksa V., Konefał R., Vosmanská M., Machová-Urdziková L. (2023). Polymer-Coated Hexagonal Upconverting Nanoparticles: Chemical Stability and Cytotoxicity. Front. Chem..

[40] He S., Xia H., Zhang J., Zhu Y., Chen B. (2017). Highly Efficient Up-Conversion Luminescence in Er^3+^/Yb^3+^ Co-Doped Na_5_Lu_9_F_32_ Single Crystals by Vertical Bridgman Method. Sci. Rep..

[41] Lashkovskaya E.I., Gaponenko N.V., Stepikhova M.V., Yablonskiy A.N., Andreev B.A., Zhivulko V.D., Mudryi A.V., Martynov I.L., Chistyakov A.A., Kargin N.I. (2022). Optical Properties and Upconversion Luminescence of BaTiO_3_ Xerogel Structures Doped with Erbium and Ytterbium. Gels.

[42] Avram D., Colbea C., Patrascu A.A., Istrate M.C., Teodorescu V., Tiseanu C. (2023). Up-Conversion Emission in Transition Metal and Lanthanide Co-Doped Systems: Dimer Sensitization Revisited. Sci. Rep..

[43] Rafique R., Baek S.H., Phan L.M.T., Chang S.-J., Gul A.R., Park T.J. (2019). A Facile Hydrothermal Synthesis of Highly Luminescent NaYF_4_:Yb^3+^/Er^3+^ Upconversion Nanoparticles and Their Biomonitoring Capability. Mater. Sci. Eng. C.

[44] Gaponenko N.V., Sudnik L.V., Vityaz P.A., Luchаnok A.R., Stepikhova M.V., Yablonskiy A.N., Lashkovskaya E.I., Shustsikava K.V., Radyush Y.V., Zhivulko V.D. (2022). Upconversion Luminescence of Er^3+^ Ions from Barium Titanate Xerogel Powder and Target Fabricated by Explosive Compaction Method. J. Appl. Spectrosc..

[45] Liu F., He X., Lei Z., Liu L., Zhang J., You H., Zhang H., Wang Z. (2015). Facile Preparation of Doxorubicin-Loaded Upconversion@Polydopamine Nanoplatforms for Simultaneous In Vivo Multimodality Imaging and Chemophotothermal Synergistic Therapy. Adv. Healthc. Mater..

[46] Zhou B., Shi B., Jin D., Liu X. (2015). Controlling Upconversion Nanocrystals for Emerging Applications. Nat. Nanotech.

[47] Campos-Gonçalves I., Costa B.F.O., Santos R.F., Durães L. (2017). Superparamagnetic Core-Shell Nanocomplexes Doped with Yb^3+^: Er^3+^/Ho^3+^ Rare-Earths for Upconversion Fluorescence. Mater. Des..

[48] Vaz-Ramos J., Cordeiro R., Castro M.M.C.A., Geraldes C.F.G.C., Costa B.F.O., Faneca H., Durães L. (2020). Supercritically Dried Superparamagnetic Mesoporous Silica Nanoparticles for Cancer Theranostics. Mater. Sci. Eng. C.

[49] Abdul Jalil R., Zhang Y. (2008). Biocompatibility of Silica Coated NaYF4 Upconversion Fluorescent Nanocrystals. Biomaterials.

[50] Wong H.-T., Tsang M.-K., Chan C.-F., Wong K.-L., Fei B., Hao J. (2013). In Vitro Cell Imaging Using Multifunctional Small Sized KGdF_4_: Yb^3+^, Er^3+^ Upconverting Nanoparticles Synthesized by a One-Pot Solvothermal Process. Nanoscale.

[51] Rostami I. (2021). Empowering the Emission of Upconversion Nanoparticles for Precise Subcellular Imaging. Nanomaterials.

[52] Yi G., Lu H., Zhao S., Ge Y., Yang W., Chen D., Guo L.-H. (2004). Synthesis, Characterization, and Biological Application of Size-Controlled Nanocrystalline NaYF_4_: Yb,Er Infrared-to-Visible Up-Conversion Phosphors. Nano Lett..

[53] Milićević B., Periša J., Ristić Z., Milenković K., Antić Ž., Smits K., Kemere M., Vitols K., Sarakovskis A., Dramićanin M. (2022). Hydrothermal Synthesis and Properties of Yb^3+^/Tm^3+^ Doped Sr_2_LaF_7_ Upconversion Nanoparticles. Nanomaterials.

[54] Zhou X., Wang Z., Li S., Shan S., Wang X. (2010). Formation and Luminescence of Sodium Rare Earth Fluoride Nanocrystals in the Presence of Chelators. J. Nanosci. Nanotechnol..

[55] Grzyb T., Przybylska D. (2018). Formation Mechanism, Structural, and Upconversion Properties of Alkaline Rare-Earth Fluoride Nanocrystals Doped with Yb^3+^/Er^3+^ Ions. Inorg. Chem..

